# What happens when we treat the “Typhoid Mary” of COVID‐19

**DOI:** 10.1002/rcr2.604

**Published:** 2020-06-25

**Authors:** Boon Hau Ng, Nik Nuratiqah Nik Abeed, Mohamed Faisal Abdul Hamid, Chun Ian Soo, Hsueh Jing Low, Yu‐Lin Andrea Ban

**Affiliations:** ^1^ Pulmonology Unit, Department of Internal Medicine, Faculty of Medicine Universiti Kebangsaan Malaysia Medical Centre Kuala Lumpur Malaysia; ^2^ Department of Anesthesiology and Intensive Care, Faculty of Medicine Universiti Kebangsaan Malaysia Medical Centre Kuala Lumpur Malaysia

**Keywords:** Computed tomography, COVID‐19, ground‐glass opacification, pneumonia, SARS‐CoV‐2

## Abstract

Severe acute respiratory syndrome coronavirus 2 (SARS‐CoV‐2) infection was declared a pandemic on 11 March 2020. We have since seen its fast spread worldwide. A likely contributing factor was the lack of symptoms of some of the carriers, making them unaware of their risk of spreading to other more vulnerable individuals. The other important finding has been the reported cases of infectivity despite lack of symptoms. We describe the SARS‐CoV‐2 pneumonia patterns in asymptomatic individuals. The common computed tomography (CT) thorax patterns found are peripheral ground‐glass opacification (GGO) with upper or lower lobe predominance. We believe screening for 2019‐novel coronavirus (COVID‐19) in high‐risk individuals may help identify the patients needing longer follow‐up.

## Graphical abstract

We describe the severe acute respiratory syndrome coronavirus 2 (SARS‐CoV‐2) pneumonia patterns in asymptomatic individuals. The common computed tomography (CT) thorax patterns found are peripheral ground‐glass opacification (GGO) with upper or lower lobe predominance. We believe screening for 2019‐novel coronavirus (COVID‐19) in high‐risk individuals may help identify the patients needing longer follow‐up.

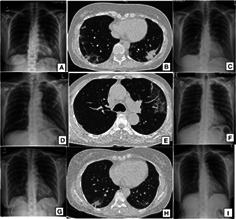

## Introduction

Severe acute respiratory syndrome coronavirus 2 (SARS‐CoV‐2) infection was declared a pandemic by the World Health Organization on 11 March 2020. 2019‐Novel coronavirus (COVID‐19) is a positive single‐stranded RNA virus belonging to the β‐coronavirus cluster. To date, two fatal respiratory diseases in human history are SARS in 2002 and Middle East respiratory syndrome (MERS) in 2012.

## Case Series

### Case 1

**Figure 1:**
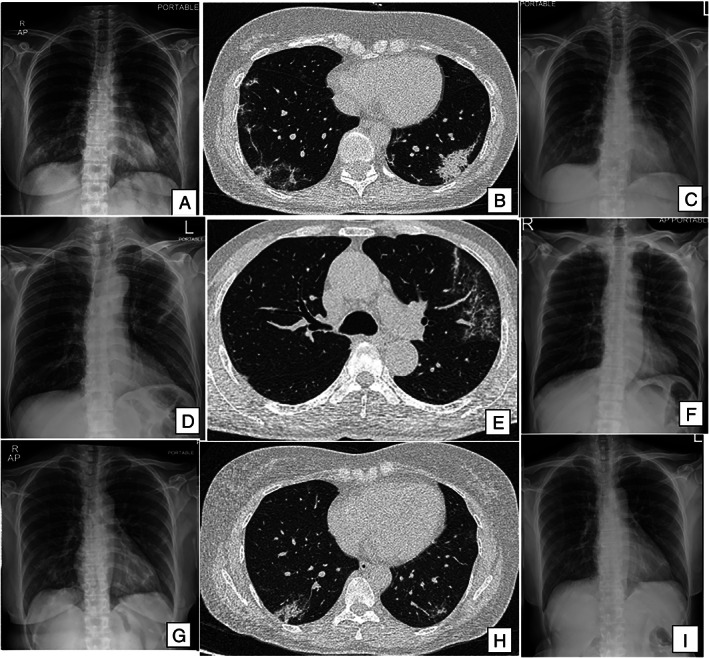
(A, D, G) Chest radiographs on admission showing various degrees of pneumonia. (B, E, H) High‐resolution computed tomography (HRCT) scans showing both ground‐glass opacification (GGO) and consolidation in the three patients. (C, F, I) Repeat chest radiographs showing resolution with treatment.

An asymptomatic 45‐year‐old woman with no known medical illness was screened for SARS‐CoV‐2 after travelling back from Scotland. She tested positive for COVID‐19 and was admitted. Her oxygen saturation was 99% on room air with a respiratory rate (RR) of 16 breaths per minute (bpm). Examination of the respiratory system was unremarkable. Chest radiograph showed bilateral lower zone air space opacities (Fig. 1A). Her Modified Early Warning Score (MEWS) was 1. We proceeded with a high‐resolution computed tomography (HRCT), which showed bilateral peripheral lower zone ground‐glass opacification (GGO) (Fig. 1B). She was treated with oral hydroxychloroquine (HCQ), azithromycin, and lopinavir/ritonavir. Chest radiograph five days later showed significant improvement of GGO (Fig. 1C). COVID‐19 real‐time reverse transcription‐polymerase chain reaction (RT‐PCR) test was detected at day 10 of illness and became negative at day 14. She was discharged well.

### Case 2

A 62‐year‐old asymptomatic man was admitted after testing positive for COVID‐19 during contact screening. He was afebrile with RR of 20 bpm and oxygen saturation of 95% on room air. Chest examination was normal. Chest radiograph showed left upper zone peripheral consolidation (Fig. 1D). HRCT thorax showed peripheral GGOs involving both the left upper and right lower lobes (Fig. 1E). He was treated with HCQ and azithromycin. The MEWS remained at 1 point throughout admission. Repeated chest radiograph at day 5 showed resolution of the GGOs (Fig. 1F).

### Case 3

A 52‐year‐old asymptomatic woman tested positive during screening after contact with a COVID‐19 individual. She had a background history of hypertension and was on perindopril 4 mg od. Clinical examination revealed a temperature of 37°C, RR of 16 bpm, and oxygen saturation of 99% on room air. Lung examination was clear. Chest radiograph showed air space opacities in the right lower zone (Fig. 1G). Her MEWS score was 2, and HRCT thorax showed evidence of bilateral lower zone peripheral GGOs (Fig. 1H). She was initiated on HCQ and azithromycin. She recovered well with interval radiological improvement (Fig. 1I, Table 1).

**Table 1:** Demographics, clinical characteristics on admission, treatment, and outcomes of three patients with COVID‐19.

Variables	Patient 1	Patient 2	Patient 3
Demographics			
Age	47	62	52
Gender	Female	Male	Female
Comorbidities	None	None	Hypertension
Smoking status	Lifelong non‐smoker	Smoker	Lifelong non‐smoker
Days from testing positive to admission	2	5	6
Clinical findings on presentation			
Temperature (°C)	36.9	37	37
Respiratory rate (bpm)	16	20	16
O_2_ saturation (in room air, %)	99	95	99
Blood pressure (mmHg)	108/64	147/87	163/97
Heart rate (bpm)	96	105	110
MEWS	1	2	1
Laboratory results			
Total white cell count (×10^9^/L)	5.1	8.0	5.1
Absolute lymphocyte count (×10^9^/L)	2.2	2.3	2.1
Absolute monocyte count (×10^9^/L)	0.4	1.2	0.5
Absolute neutrophil count (×10^9^/L)	2.4	4.4	2.4
Platelet (×10^9^/L)	285	467	414
LDH (U/L)	335	349	306
C‐reactive protein (mg/dL)	0.21	1.57	2.13
D‐dimer (ng/mL)	0.28	0.27	1.89
Ferritin (μg/mL)	182.72	—	103.67
Fibrinogen (g/L)	2.5	5.7	2.7
Procalcitonin (ng/dL)	<0.02	<0.02	<0.02
Radiological imaging			
Chest radiograph	Left upper zone peripheral consolidation	Left upper zone peripheral consolidation	Air space opacities in the right lower zone
HRCT	Bilateral lower zone peripheral GGOs	Peripheral GGOs involving both the left upper and right lower lobes	Bilateral lower zone peripheral GGOs
Treatment and outcome			
Drug therapy	HCQ	HCQ	HCQ
	400 mg bd Day 1	400 mg bd Day 1	400 mg bd Day 1
	200 mg bd Days 2–10 azithromycin	200 mg bd Days 2–5 azithromycin	200 mg bd Days 2–10 azithromycin
	500 mg od Day 1	500 mg od Day 1	500 mg od Day 1
	250 mg od Days 2–5 lopinavir/ritonavir	250 mg od Days 2–5	250 mg od Days 2–5
	400/100 mg bd Days 1–10		
Length of hospital stay (days)	14	10	16
Repeated COVID‐19 RT‐PCR test	Positive at day 10. Negative at days 13 and 14	Negative at days 10 and 13	Negative at days 10 and 13

bd, Twice daily; bpm, breaths per minute; COVID‐19, 2019‐novel coronavirus; GGO, ground‐glass opacification; HCQ, hydroxychloroquine; HRCT, high‐resolution computed tomography; LDH, lactate dehydrogenase; MEWS, Modified Early Warning Score; RT‐PCR, reverse transcription‐polymerase chain reaction.

## Discussion

Patients with COVID‐19 have a wide range of symptoms with the majority presenting with an influenza‐like illness of fever, cough, rhinorrhoea, and sore throat [1]. Less common non‐respiratory symptoms include diarrhoea, anosmia, pericarditis, Guillain–Barré syndrome, or acute necrotizing encephalopathy [2, 3]. Observational studies have reported up to 12% of transmissions occurring before the onset of clinical symptoms [4].

Published data have shown that up to 75% of positive SARS‐CoV‐2 patients can be asymptomatic [5, 6]. All three patients in our case series displayed no symptoms of COVID‐19 disease despite showing radiological evidence of pneumonia. With the absence of symptoms, these patients may not have voluntarily come forward for screening. Screening based on symptoms alone may miss a significant amount of asymptomatic COVID‐positive patients. Targeted screening in these patients have allowed immediate isolation from the community and initiation of treatment which may have prevented disease progression and achieved earlier recovery.

The common laboratory abnormalities are lymphopenia, increased lactate dehydrogenase, and elevated C‐reactive protein [7]. These parameters are non‐specific and their clinical usefulness is limited. These abnormalities may be related to the cytokine storm in COVID‐19 and the changes are similar to patients with SARS and MERS.

We performed the initial imaging tool of chest radiograph which showed varying locations of pneumonia (Fig. 1). As COVID‐19 is a novel disease, we chose to proceed with a HRCT to allow further clarification on the extent of the pathologies. We found GGO with peripheral distribution and upper and lower lobes predominance in our patients. Our findings are similar with the known initial computed tomography (CT) manifestations of COVID‐19 [8]. The most common reported CT findings are isolated GGO or a combination of GGO and consolidative opacities [8]. Less common findings include interlobular septal thickening, bronchiectasis, pleural effusion, pleural thickening, and subpleural involvement, cavitation and lymphadenopathy [8]. On average, CT findings are most prominent on day 10 of illness and improve after day 14 [9].

In a study including nine hospitals from the region of Hubei, 16 (11.7%) out of 117 patients died from COVID‐19 disease [10]. A systematic review found the mortality rate to be higher from studies in Wuhan, the epicentre of this outbreak, with lower prevalence of death among patients from outside Wuhan. They also found that older patients with comorbidities may have a more severe impact from the disease [11].

All three patients in our report had a negative RT‐PCR test by day 13 of illness. A study reported a median of 36 days from the date of symptoms to the date of first negative RT‐PCR test, with the longest reported at 45 days from symptom onset [10]. More recent studies have shown that pharyngeal virus shedding does not equate to infectivity. This shedding appears to peak at day 4 and is the highest during the first week of symptoms [12].

This case series describes SARS‐CoV‐2 pneumonia patterns in asymptomatic individuals. The common CT thorax patterns found are peripheral GGO with upper or lower lobe predominance. We believe screening for COVID‐19 in high‐risk individuals is important to see the extent of pulmonary damage and assess the possible need for long‐term lung function follow‐up.

### Disclosure Statement

Appropriate written informed consent was obtained for publication of this case series and accompanying images.

At the time this report was accepted for publication, the authors declared that the patients in this report had not been included in any previously published report on COVID‐19 that they had authored.
